# Culturally Adapted Mental Health Education Programs for Migrant Populations: A Scoping Review

**DOI:** 10.3390/ijerph23010072

**Published:** 2026-01-02

**Authors:** Shaima Ahammed Thayyilayil, Sophie Yohani, Lisa Cyuzuzo, Megan Kennedy, Bukola Salami

**Affiliations:** 1 Faculty of Education, University of Alberta, 11210 87 Avenue, Edmonton, AB T6G 2T9, Canada; thayyila@ualberta.ca (S.A.T.); lcyuzuzo@ualberta.ca (L.C.); 2 Geoffrey & Robyn Sperber Health Sciences Library, University of Alberta, 11405 87 Avenue, Edmonton, AB T6G 1C9, Canada; mrkenned@ualberta.ca; 3 Department of Community Health Sciences, Cumming School of Medicine, University of Calgary, Calgary, AB T2N 1N4, Canada; oluwabukola.salami@ucalgary.ca

**Keywords:** mental health education, migrants, cultural adaptation, community-based interventions, scoping review

## Abstract

Migrant populations drive Canada’s demographic expansion, making their successful integration a national priority. However, research has consistently shown that refugees and immigrants experience declining mental health and encounter significant barriers to accessing culturally appropriate mental health support. This scoping review examined the breadth of evidence on culturally adapted mental health education (MHE) programs for migrant populations, including those that integrate traditional and complementary healing practices, and their effectiveness. Systematic searches across six databases (Medline, EMBASE, PsycINFO, Global Health, CINAHL, and Scopus) identified 4075 peer-reviewed articles, with 28 studies meeting inclusion criteria. These included mental health education and awareness programs that integrate psychoeducation and skill-building. Inclusion criteria required cultural adaptation of programs through one or more approaches such as language modification, culturally adapted content, community-based delivery, or integration of traditional and complementary healing practices. Thematic analysis of the programs revealed seven key themes characterizing effective MHE programs: cultural adaptation and sensitivity, addressing unique migration-related stressors, integration of traditional and Western approaches, use of theoretical frameworks and evidence-based practices, rigorous evaluation methodologies, application of holistic frameworks, and community-based peer support models. Programs predominantly utilized psychoeducation and culturally adapted interventions, with common theoretical frameworks including cognitive–behavioral therapy and the PRECEDE–PROCEED model. Across the reviewed studies, program evaluations reported positive outcomes including increased mental health literacy, reduced stigma, enhanced coping skills, and decreased depression, anxiety, and PTSD symptoms, suggesting that culturally adapted MHE programs are acceptable and feasible interventions for migrant populations.

## 1. Introduction

Recent shifts in migration patterns pose significant challenges for mental health systems in host countries such as Canada, where diverse newcomer populations face barriers accessing mental health services [1,2]. The increasing scale of global conflicts, forced displacements, socio-economic instability and political persecution has led to increases across different categories of migrants including refugees, asylum seekers, voluntary migrants, and undocumented populations. Members of these groups experience a range of psychosocial stressors that shape mental health outcomes in ways that are not easily recognized or addressed by healthcare providers unfamiliar with migration-specific trauma and acculturation processes [3]. Forced migrants often endure traumatic exposures before departure such as war-related violence, interpersonal assaults, or prolonged deprivation; these experiences set the stage for heightened susceptibility to disorders including post-traumatic stress disorder (PTSD), depression, and anxiety long after resettlement [4,5]. Economic migrants, on the other hand, may face different but equally significant stressors including family separation, financial insecurity, employment exploitation, and social isolation, which can manifest as adjustment disorders, chronic stress responses, and identity-related psychological distress. Both groups encounter additional post-migration challenges such as language barriers, cultural dissonance, discrimination and navigating complex immigration systems, creating layered vulnerabilities that require nuanced understanding and tailored intervention approaches rather than standardized mental health responses designed for settled populations [6].

The complex mental health needs of migrants underscore the importance of developing accessible and culturally responsive interventions that can bridge the existing service gaps and facilitate their successful integration and psychological wellbeing. Eurocentric, mainstream mental health services often prove insufficient in addressing the unique combination of cultural barriers, stigma, and systemic obstacles that prevent migrants from accessing care [7]. It is in this context that mental health education (MHE) programs emerge as a promising strategy to address these challenges by focusing on prevention, early intervention, and community-based support rather than relying on crisis-response approaches in traditional clinical models. Such programs can enhance mental health literacy, reduce stigma, build coping skills, and create pathways to appropriate care while being culturally responsive and, where appropriate, integrating cultural healing approaches [8,9].

The potential for MHE programs is particularly compelling when they incorporate cultural healing practices, including traditional knowledge and indigenous healing approaches from migrants’ cultural traditions and countries of origin [10,11,12]. Such approaches recognize that mental health and wellbeing are understood and addressed differently across cultures, and that effective interventions must honor these diverse perspectives while providing evidence-based support. By integrating traditional knowledge with contemporary mental health education, programs can create culturally safe spaces that feel familiar and accessible to migrant communities while building bridges to mainstream services when needed.

Given the growing recognition of the importance of culturally safe mental health programs, there is a critical need to synthesize existing evidence on culturally responsive MHE programs for migrant populations. Also, the field lacks a comprehensive understanding of effective cultural adaptation strategies for mental health interventions serving migrant populations. While some programs have begun integrating traditional and indigenous healing practices alongside evidence-based approaches, limited synthesis exists regarding the spectrum of cultural adaptation approaches—from surface-level modifications (e.g., language translation, culturally appropriate examples) to deep integration of cultural healing practices—and their implementation and effectiveness across different migrant contexts.

To address this knowledge gap, this scoping review systematically examined culturally adapted mental health education programs for migrant populations. The review had three specific objectives:

1. To map the scope and characteristics of existing MHE programs, including target populations, settings, and types of interventions.
2. To identify the range of cultural adaptation strategies employed across programs.
3. To examine reported program outcomes and evaluation findings.

By mapping the current evidence base, this review sought to identify promising practices, illuminate critical gaps in existing knowledge, and provide evidence-based guidance for developing more effective, culturally responsive mental health education interventions that can better serve the complex needs of migrant communities.

## 2. Method

### 2.1. Design

This scoping review utilized the Joanna Briggs Institute (JBI) methodological guidelines for scoping reviews [13,14] and the PRISMA guidelines for designing, conducing and reporting scoping reviews (PRISMA-ScR) [15]. A completed PRISMA-ScR checklist is provided in the Supplementary Materials to demonstrate adherence to reporting standards. The team collaborated with a health science librarian (MK) on this study to refine the search strategy and to develop a question that was appropriately broad and aligned with the research goal. As such this scoping review was guided by the following research question:

What is known about culturally adapted mental health education programs for migrant populations, including their design, implementation strategies, cultural adaptation approaches, and effectiveness?

Three sub-questions guided this inquiry:

1. What types of cultural adaptation strategies are employed in these programs (including language modifications, delivery models, integration of cultural values, and traditional healing practices)?
2. What are the key components, theoretical frameworks, and delivery approaches of culturally responsive programs?
3. What outcomes and evidence of effectiveness have been reported across different migrant populations and settings?

### 2.2. Search Strategy

A comprehensive search strategy was developed using the Population–Concept–Context (PCC) framework. The following databases were searched from inception to February 29th, 2024: Medline (1946–present), EMBASE (1974–present), PsycINFO (1806–present), and Global Health (1910–present) via OVID; CINAHL (1936–present) via EBSCOhost; and Scopus (1976–present).

The search strategy incorporated three main concepts aligned with the PCC framework:

1. Population: Immigrants, migrants, refugees, asylum seekers, and undocumented migrants.
2. Concept: Mental health education (to capture diverse expressions of mental health literacy programming including psychoeducation and mental health skills building).
3. Context: Cultural adaptation strategies, including culturally safe, culturally sensitive, and culturally adapted delivery approaches, as well as traditional and complementary healing practices (traditional medicine, spiritual healing, faith-based approaches, complementary therapies).

The cultural adaptation component of the search was comprehensive, including terms related to cultural competency and sensitivity, traditional and complementary healing practices (e.g., spiritual healing, faith-based approaches, complementary therapies, yoga, breathing exercises, etc.), and language/religious adaptation. This broad search strategy was intentionally designed to capture the spectrum of cultural adaptation approaches, from surface-level modifications to deep integration of traditional, indigenous and complementary healing practices. The search included extensive terms for traditional medicine and complementary therapies (see Appendix A), though programs were not required to incorporate traditional knowledge systems to be included. Keywords and subject headings (including MeSH terms where available) were combined using Boolean operators. No publication date or language restrictions were applied to maximize search sensitivity. An example search strategy (Ovid MEDLINE) is provided in Appendix A.

### 2.3. Inclusion and Exclusion Criteria

Studies were included if they:

1. Focused on migrants (including refugees, immigrants, undocumented migrants, and asylum seekers).
2. Described mental health education programs, including psychoeducation, mental health literacy, mental health awareness, and skill-building.
3. Demonstrated cultural adaptation or cultural responsiveness through one or more of the following approaches: a. language translation or bilingual delivery; b. culturally adapted content (e.g., metaphors, or case vignettes); c. incorporation of cultural values, beliefs, or worldviews; d. community-based, peer-led, or culturally matched delivery models; e. use of cultural idioms of distress or local mental health terminology; f. integration of traditional medicine, complementary healing practices, spiritual approaches, or faith-based healing.
4. Were conducted in migrant-receiving countries.
5. Were published in English.

For criterion 2, programs qualified if they included educational components aimed at enhancing mental health awareness, knowledge, skills, or help-seeking behaviors, whether delivered as a psychoeducational program or as part of therapeutic interventions.

Studies were excluded if they:

1. Focused on populations other than migrants.
2. Addressed physical health without mental health as a central focus.
3. Focused solely on clinical interventions without any mental health awareness, psychoeducation, or skill-building elements.
4. Did not demonstrate any form of cultural adaptation, cultural tailoring, or cultural responsiveness to migrant populations.
5. Were non-peer reviewed publications (e.g., book chapters).

### 2.4. Screening Process and Data Extraction

**Figure 1:**
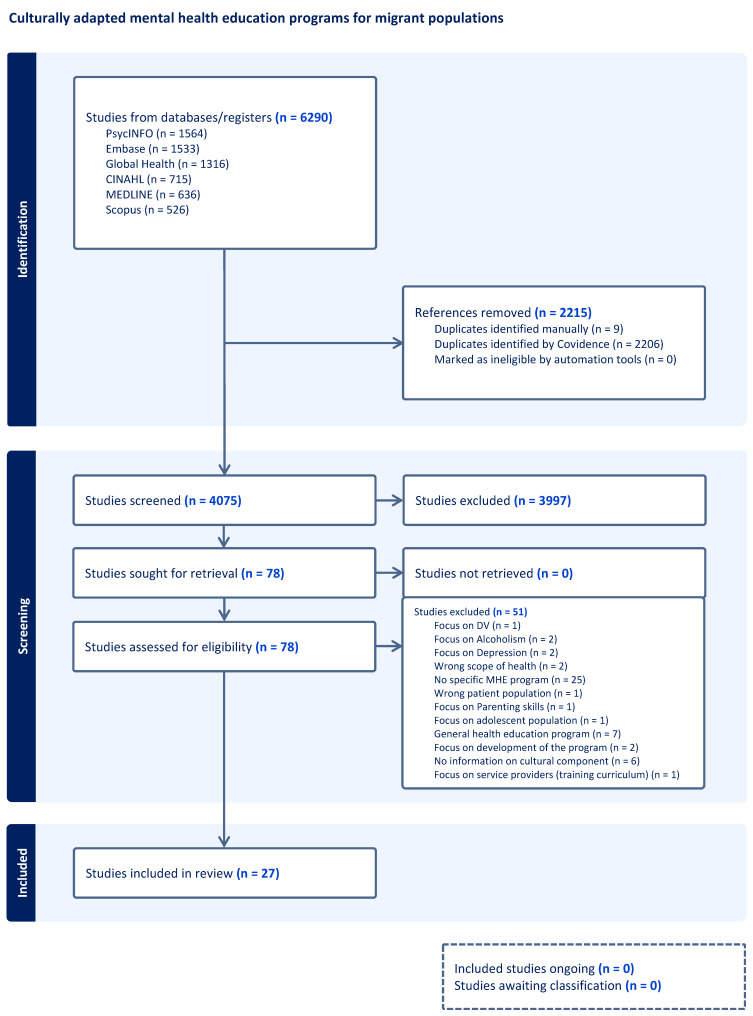
PRISMA flow diagram of the search process. Note. PRISMA = Preferred Reporting Items for Systematic Reviews and Meta-Analyses.

The search yielded 4075 articles, with duplicates removed using Covidence systematic review management software. Three researchers independently screened titles and abstracts, with conflicts resolved through discussion and consensus. A total of 78 studies proceeded to full-text review by the same three researchers, from which 27 studies met final inclusion criteria (Figure 1). An additional article was included after a systematic reference screening of all retrieved full texts, resulting in 28 studies. We also completed a grey literature search using the terms used in our database search in April 2024. Grey literature was identified through targeted Google searches, including Google Scholar, and relevant migrant-serving health websites searches both internationally and in Canada. The grey literature search resulted in 4 documents that met inclusion criteria.

### 2.5. Charting the Data

A data extraction chart was created to capture key details of the selected 28 articles including publication information (title, authors, publication year), participant demographics (country of origin, gender, age, sexual orientation), study characteristics (methodology, location), mental health program details (content, structure, theoretical framework), target population, cultural adaptations, traditional knowledge integration, key findings, study limitations, and recommendations for future research and practice.

Data extraction was performed by researcher (ST) and independently verified by two researchers (LC, SY) to ensure accuracy and completeness. Extracted data included study characteristics, population details, intervention features, cultural adaptation strategies, and reported outcomes. Any discrepancies or uncertainties identified during verification were resolved through discussion and consensus among all three researchers. The extracted data were organized in Appendix B and synthesized narratively in the Results Section 3. A condensed data extraction table is provided in Appendix B and Appendix C.

### 2.6. Collating, Summarizing and Reporting the Results

Extracted data were synthesized and then subjected to a thematic analysis to identify patterns, commonalities, and differences across studies aligned with the research question. The synthesis focused on key aspects of program design, development, implementation approaches, content characteristics, and cultural adaptations. Themes were developed through an iterative process of data review, with researchers collaboratively refining categories until consensus was reached on the final list of themes. In the following section, the results are presented by reporting a summary of the key characteristics across all studies, followed by program-specific characteristics. A second section reports on the main themes that characterize the essential components and recommended practices for developing culturally adapted MHE programs for migrant populations.

## 3. Results

### 3.1. Key Characteristics of Studies

A total of 28 studies met the inclusion criteria for this scoping review and are summarized in Table 1. The majority of studies were conducted in the United States (n = 12), followed by Australia (n = 4), Turkey (n = 2), and Germany (n = 2), with single studies from the United Kingdom, Canada, Sweden, Netherlands, Kenya, Pakistan, and Uganda. All included studies were published between 2010 and 2024, with an overwhelming majority (n = 21) published in the past four years. Study designs included qualitative studies (n = 15), mixed methods (n = 6), quantitative studies (n = 4), and pilot/feasibility studies (n = 3). Sample sizes ranged from 5 to 1485 participants. The studies included diverse participant types: community members (n = 18), clients/service users (n = 12), service providers (n = 8), community leaders (n = 5), and family members (n = 3).

Among the included studies, several evidence-based interventions demonstrated adaptability across different migrant populations and settings. Notably, four interventions were implemented in multiple studies: Mental Health First Aid (MHFA) in 2 studies, Problem Management Plus (PM+) in 3 implementations, Self-Help Plus (SH+) in 2 implementations, and ALMA (Amigas Latinas Motivando el Alma) in 2 studies. These interventions showed consistent cultural adaptation strategies including language translation, community-based delivery, peer facilitation, and integration of cultural values. A summary of these interventions with multiple implementations, including populations served, settings, cultural adaptations, and key outcomes, is provided in Appendix D. The following sections provide detailed characteristics of all included studies and programs.

### 3.2. Population and Program Characteristics

The MHE programs served diverse migrant populations. The most frequently targeted populations were Latino/Hispanic immigrants (n = 8), Arabic-speaking refugees (n = 6), and various African populations including sub-Saharan African refugees (n = 7). Filipino migrants (n = 1), Bhutanese refugees (n = 2), and Syrian refugees (n = 2) were also represented.

Programs predominantly targeted adult populations (n = 16), with some focusing on specific age groups including youth (n = 2), older adults (n = 1), or family units spanning multiple generations (n = 2). Gender distribution showed mixed-gender programs (n = 13), women-only programs (n = 8), men-only programs (n = 1), and programs explicitly inclusive of gender-diverse populations (n = 2).

### 3.3. Program Structure and Delivery Modalities

Program duration varied considerably, ranging from single-session interventions to 14-week programs. The most common format was group-based delivery (n = 18), with individual sessions (n = 6) and family-based approaches (n = 4) also represented. Session length typically ranged from 90 min to 3 hours. Delivery modalities included in-person group sessions (n = 20), online/virtual platforms (n = 4), self-paced digital modules (n = 3), and hybrid approaches (n = 1). Programs were implemented across various settings including community centers (n = 12), healthcare facilities (n = 8), religious venues (n = 5), and educational institutions (n = 3).

### 3.4. Program Content and Focus Areas

The programs addressed diverse mental health concerns. General mental health promotion and literacy were the most common focus (n = 8), followed by trauma and Post-Traumatic Stress Disorder (PTSD) interventions (n = 5), and programs addressing racism-related stress and discrimination (n = 4). Other focus areas included depression and anxiety management (n = 4), settlement and integration challenges (n = 3), and social support enhancement (n = 3). Specialized programs targeted specific populations such as postpartum women (n = 2) and survivors of sexual violence (n = 1).

### 3.5. Theoretical Frameworks and Approaches Used in Programs

The studies employed various theoretical frameworks to guide program development and implementation. Cognitive–behavioral therapy principles were most commonly utilized (n = 8), followed by community-based participatory research approaches (n = 6). Cultural adaptation frameworks, including Bernal’s framework [16] and other systematic adaptation models, were employed in five studies. Additional frameworks included social cognitive theory (n = 3), trauma-informed care principles (n = 3), and positive psychology approaches (n = 2). Several studies (n = 6) combined multiple theoretical approaches.

### 3.6. Cultural Adaptations and Traditional Knowledge Integration in Programs

All included programs incorporated cultural adaptations, though the depth and approach varied considerably. Surface-level adaptations were universal and included language translation, culturally appropriate imagery, and modified delivery methods. Deep-level adaptations, found in 18 studies, involved integration of cultural values, traditional healing practices, and community-specific worldviews. Traditional knowledge integration was explicitly reported in 15 studies. Common approaches included incorporation of religious and spiritual practices (n = 12), traditional healing ceremonies and rituals (n = 8), cultural storytelling and narratives (n = 7), and integration of indigenous explanatory models of mental health (n = 6). Several programs (n = 4) included traditional healers as co-facilitators or consultants.

### 3.7. Program Outcomes and Effectiveness

Most studies (n = 22) reported positive outcomes, though the quality of evidence varied given the predominance of pilot and feasibility studies. Commonly reported improvements included increased mental health literacy (n = 16), reduced stigma toward mental health services (n = 14), enhanced coping skills (n = 12), and increased help-seeking behaviors (n = 10). Programs addressing trauma showed reductions in PTSD symptoms (n = 4), while those targeting depression reported decreased symptom severity (n = 3). Cultural adaptation appeared to enhance program acceptability, with 24 studies reporting high participant satisfaction and engagement. Programs incorporating traditional knowledge elements showed particularly strong community acceptance and retention rates, though comparison groups were rarely employed.

### 3.8. Program Implementation Challenges and Facilitators

Studies identified several implementation challenges including recruitment difficulties (n = 12), language barriers (n = 8), transportation and accessibility issues (n = 7), and stigma-related barriers (n = 6). COVID-19 pandemic restrictions affected seven studies, leading to adaptations in delivery methods. Facilitating factors included community partnership and co-design approaches (n = 18), peer-led facilitation (n = 10), flexible scheduling (n = 8), and provision of childcare or transportation support (n = 6). Programs that engaged community leaders and cultural brokers reported enhanced community buy-in and sustainability.

### 3.9. Key Features of Culturally Adapted Mental Health Education Programs

A thematic analysis of the 28 studies and 4 grey literature sources revealed seven main themes that characterize the essential components and recommended practices for developing culturally adapted MHE programs for migrant populations (Table 2). The themes included: cultural adaptation and sensitivity, addressing unique migration-related stressors and challenges, integration of traditional and Western approaches, use of theoretical frameworks and evidence-based practices, evaluation methodologies, application of holistic frameworks, and use of community-based peer support models.

#### 3.9.1. Cultural Adaptation and Sensitivity

A major theme across the reviewed papers is the importance of cultural adaptation and sensitivity in the development and implementation of MHE programs for refugee and immigrant populations. Interventions that are tailored to align with the cultural beliefs, values, and practices of the target population have been shown to be more engaging, acceptable, and effective [17,18,19]. This involves adapting the intervention content, delivery methods, and materials to be culturally appropriate and relevant. For example, Akhtar and colleagues [17] conducted a cultural adaptation of a low-intensity group psychological intervention for Syrian refugees in Jordan and Turkey, making modifications to the language, metaphors, content, and context based on input from local stakeholders and cognitive interviews with the target population. Additionally, the Women’s Health manual (Hong Fook Mental Health Association in Canada) included culturally meaningful activities like tapestry-making and used culture-specific objects [20]. Strategies for enhancing cultural sensitivity include involving community members, leaders, and organizations in intervention development and delivery [21,22] using culturally informed examples, metaphors, proverbs, and case studies [23,24] and enhancing providers’ cross-cultural understanding, empathy, and humility through training and interactions with community members [22,23]. However, the Group PM+ program [25], a grey literature source, cautions that it is important to challenge particular cultural beliefs or practices that are harmful (e.g., “rape is the fault of the victim” or “beating the spirit out heals mental illness”) when delivering MHE programs.

#### 3.9.2. Addressing Unique Migration-Related Stressors and Challenges

MHE programs in this review highlighted the need to address the unique stressors and challenges faced by migrant populations. These may include pre- and post-migration traumas, acculturation stress, discrimination, social isolation, and barriers to accessing mental health services [23,24,26]. Interventions often provide psychoeducation on common mental health issues in these populations, such as PTSD, depression, and anxiety [23,27], and teach coping skills and strategies to manage stressors [23,24]. Key interventions include grounding, problem-solving, behavioral activation, strengthening social support, and emotional regulation [3,27,28,29]. For instance, Sabri and colleagues [26] developed the BSHAPE intervention for immigrant survivors of cumulative trauma, which comprehensively assessed their trauma experiences and current safety needs while providing components to reduce the impact of trauma on mental health, stress response, and HIV/STI risk.

#### 3.9.3. Integration of Traditional and Western Approaches

The integration of traditional healing practices and cultural beliefs with evidence-based Western approaches is another important theme in the development of culturally sensitive MHE programs. This involves incorporating cultural expressions, concepts of distress, healing practices, and beliefs [22,23] and combining evidence-based techniques like cognitive–behavioral therapy (CBT) with traditional practices such as prayer and meditation [24]. For example, Omidian [30] incorporated Islamic teachings and Afghan cultural metaphors into a psychosocial wellness training program for Afghan teachers to ensure engagement and acceptability among participants. Interventions may also discuss cultural practices alongside information on accessing mainstream services to balance the two approaches [21,22].

#### 3.9.4. Theoretical Frameworks and Evidence-Based Practices

The interventions described in the reviewed literature were often grounded in established theoretical frameworks and evidence-based practices, which were adapted to suit the specific cultural context. These included social cognitive theory and modeling of coping behaviors [27], culture-centric narrative models [27], guidelines for culturally sensitive CBT with refugees [23], trauma recovery models and trauma-informed approaches [23,26] and social constructivism and intersectionality frameworks [22]. Evidence-based practices such as mindfulness-based stress reduction, psychoeducation, and problem-solving were also incorporated into the interventions [26,30,31].

#### 3.9.5. Evaluation Methodologies

The studies employed various evaluation methodologies to assess the feasibility, acceptability, and effectiveness of the interventions. Pre-post designs were commonly used [26,32,33], with some studies also including follow-up assessments [26,32]. Randomized controlled trials comparing the intervention to a control group were also conducted in some studies [23,27]. Mixed-method approaches, incorporating both quantitative and qualitative data, were utilized to gain a comprehensive understanding of the interventions’ impact [26,30,34]. Outcome measures typically included assessments of mental health literacy, stigma, symptoms, and attitudes [21,23,27]. Most studies suggested MHE programs increased participant mental health and well-being. The results indicated increased coping skills and sense of social support, reduced mental health stigma, amelioration of depression, anxiety and PTSD symptoms, increased recognition of mental illness symptoms, enhanced communication and openness to mental healthcare services [27,35,36,37].

#### 3.9.6. Application of Holistic Frameworks

Some of the literature acknowledged the importance of applying an intersectional approach to understand the multifaceted aspects and complex interplay of factors (e.g., race, gender, and immigration status) that shape the mental health needs of refugee and immigrant populations. Programs were designed to address the physical, psychological, spiritual, social, and environmental determinants of health and wellbeing [20,25,38]. For example, the mind–body connection is addressed in the HIAS program [38]. Also, the BSHAPE Intervention [26] highlighted how the cumulative trauma experiences of Black immigrant women intersect with racism, discrimination, sexism, classism, and other structural and situational stressors, necessitating a nuanced understanding and approach to addressing violence and related health issues. Incorporating an intersectional lens in MHE can help to develop more comprehensive and effective interventions that address the multiple, intersecting challenges faced by these diverse populations. It also emphasizes the importance of equity, access, and social justice.

#### 3.9.7. Community-Based Peer Support Models

Most of the programs emphasized community partnership and applied a community-based approach where briefly trained lay providers like peer leaders or community workers delivered the programs [37,39]. This enhances engagement, trust and social support, and accessibility. As noted in [38] “community members can guide other newcomers in accessing culturally appropriate psychosocial care that promotes healing, safety, and resilience in affected communities.” (p. 6). For instance, the ALMA pilot Promotora intervention and Islamic Trauma Healing program were developed in close partnership with target communities and both employed peer or community facilitators [39,40]. This approach empowers community members and cultural advisory groups as co-designers and facilitators, not just passive program recipients.

## 4. Discussion

This scoping review has synthesized findings from the existing literature on culturally adapted mental health education programs for migrant populations. We identified 28 studies and 4 grey literature sources focused on MHE programs for diverse populations of adult migrants in over 11 countries. These sources provided information on key program components, outcomes and evaluation of MHE programs that promote refugee and immigrant mental health and wellbeing. The data suggests that culturally adapted MHE programs serve as acceptable, feasible, and potentially effective interventions for migrant populations, with participants experiencing enhanced mental health, shifts in negative attitudes and beliefs about mental health, increased willingness to access services, and a strengthened sense of empowerment and connection.

The findings highlight the necessity of transitioning from superficial cultural adaptation to substantive cultural safety frameworks in MHE programs and mental health service delivery. Cultural safety, a framework originating from Maori nursing scholarship [41,42] moves beyond superficial cultural adaptations to critically interrogate the power imbalances and systemic discrimination that affect mental health outcomes for marginalized populations [43,44]. Central to this framework is the dismantling of hierarchical models that position Western biomedical knowledge as inherently superior to local explanatory frameworks of distress. The reviewed MHE programs demonstrate how service providers can engage dialogically with migrant people allowing biomedical perspectives to coexist alongside diverse explanatory models of distress rooted in spiritual or traditional knowledge systems common in many migrant communities. Such dialogical and relational approaches move beyond mere tolerance of diverse health beliefs toward genuine epistemic humility which recognizes multiple ways of understanding mental distress hold validity and can complement one another in therapeutic contexts. This approach is consistent with literature that calls for cultural safety in mental health services as a means of achieving health equity [45].

The thematic analysis revealed seven key components characterizing effective mental health education (MHE) programs: cultural adaptation and sensitivity, addressing unique migration-related stressors and challenges, integration of traditional and Western approaches, use of theoretical frameworks and evidence-based practices, rigorous evaluation methodologies, application of holistic frameworks, and community-based peer support models. These components align with expanded definitions of mental health literacy that extend beyond knowledge acquisition to include reducing stigma and improving help-seeking behaviors [46,47]. The MHE programs reviewed integrated surface-level adaptations (language translation, culturally appropriate imagery) with deeper adaptations involving cultural values, traditional healing practices, and community-specific worldviews—a distinction consistent with frameworks differentiating between superficial and deep-structure cultural adaptations in health interventions [48,49]. Notably, fifteen studies explicitly incorporated traditional knowledge, including religious and spiritual practices, traditional healing ceremonies, cultural storytelling, and indigenous explanatory models of mental health, reflecting growing recognition of the importance of integrating multiple knowledge systems in mental health care for diverse populations [50,51].

Improvements in common mental health concerns such as depression, anxiety, and PTSD symptoms were frequently reported in MHE programs across the reviewed studies, consistent with meta-analytic evidence supporting the efficacy of culturally adapted mental health interventions [52]. Participants in the MHE programs appeared to gain understanding and language for mental health symptoms and challenges, with 16 studies reporting increased mental health literacy and 14 documenting reduced stigma toward mental health services. The peer/ community facilitator led components appeared effective in normalizing symptom discussion and framing help-seeking as consistent with community values- findings that align with research demonstrating the effectiveness of peer support in reducing mental health stigma and increasing service utilization among marginalized groups [53]. This community dimension may have added an implicit layer of safety by reframing learning as internal community dialogue rather than external instruction, potentially contributing to participants’ increased trust in the programs.

The holistic frameworks employed by many MHE programs acknowledged the intersection of physical, psychological, social, and spiritual dimensions of well-being, reflecting diverse cultural conceptualizations of health, including indigenous and non-Western frameworks, that challenge biomedical reductionism, e.g., [54,55]. These programs were designed to address multiple determinants of health and wellbeing, recognizing that pre- and post-migration traumas, acculturation stress, discrimination, social isolation, and structural barriers such as racism, poverty, and immigration status fundamentally shape migrant mental health [56,57]. The literature emphasizes that investing in mental health interventions for marginalized populations that address social determinants can potentially improve not only mental but also physical and social health outcomes, e.g., [58]. The reviewed programs commonly taught coping skills and strategies to manage stressors, including grounding, problem-solving, behavioral activation, strengthening social support, and emotional regulation approaches [23,24,28,38,59], consistent with contextually adapted cognitive–behavioral interventions for refugee populations [60,61]. Additionally, programs that applied ecological frameworks emphasizing family and community interventions alongside individual skill-building appeared effective, an approach consistent with ecological systems theory [62,63]. These programs included conjoint family sessions to process migration stressors and engaging local faith and cultural groups in combating stigma and mobilizing social support.

The community-based participatory approach emerged as a critical facilitator of program success in the reviewed MHE programs. Most programs emphasized community partnership, with trained peer leaders or community workers delivering interventions alongside mental health professionals. This co-facilitated model, where mental health educators work alongside respected community figures and cultural advisory groups, embeds psychoeducational content within culturally resonant narratives while also giving space to communal coping frameworks [64] and fostering trust toward unfamiliar healthcare systems—a critical consideration given documented histories of medical mistrust among marginalized communities [65]. This approach appeared to enhance engagement, trust, social support, and accessibility which are outcomes that are well-documented in the community-based participatory research (CBPR) literature [66,67].

Several MHE programs in the review described potential for sustainability and scalability through train-the-trainer models, partnerships with ethnic media for mental health literacy dissemination, and advancement of newcomer leadership in research, service planning, and policy spheres. The modular curricula, peer-led facilitation, and digital platforms identified in several studies may offer pathways to extend reach without compromising cultural congruence. However, scaling these models likely requires careful balancing of fidelity to core mental health literacy objectives with adaptability to diverse local contexts and evolving migrant demographics—a tension well-recognized in the cultural adaptation of mental health interventions, e.g., [68].

### 4.1. Limitations and Implications for Research and Practice

The strength of this review lies in its comprehensive mapping of MHE programs across various settings and populations, drawing from both primary and grey literature. The review included independent screening and selection by three researchers ensuring consensus of selected articles. The study has provided a detailed understanding of culturally adapted MHE programs, their key components, cultural adaptation strategies, implementation approaches, and reported outcomes across diverse migrant populations and settings. However, there are several limitations to this review that warrant acknowledgment and suggest directions for future research.

While our search strategy was comprehensive and systematic, combining cultural adaptation terms with MHE terms across six databases, we acknowledge that some relevant studies may not have been captured. The field of culturally adapted mental health interventions for migrants is rapidly evolving, and studies published after our search date (29 February 2024) were not available for inclusion. Notable examples include recent implementations of established intervention platforms such as Problem Management Plus [69] and Step-by-Step [70] that represent emerging evidence in this area. Additionally, studies may have been missed due to indexing patterns. Studies indexed primarily under specific intervention platforms (e.g., “Problem Management Plus,” “Self-Help Plus,” “Integrative ADAPT Therapy”) without sufficient keywords representing cultural adaptation in their titles or abstracts, may not have been captured by our search filters. This represents a methodological challenge in scoping reviews: more specific search terms maintain focus but may miss relevant studies indexed differently. A complementary search strategy combining cultural adaptation terms with specific intervention names (e.g., “Problem Management Plus” AND “cultural adaptation”) could have captured additional MHE programs such as PM+ [71,72], Self-Help Plus [73,74], and Integrative ADAPT Therapy [75].

Another methodological limitation relates to quality appraisal of the included studies. As a scoping review focused on mapping the breadth of available evidence rather than synthesizing effectiveness, formal risk of bias assessment was not undertaken. This aligns with the methodological framework for scoping reviews recommended by Arksey and O’Malley [76]. The framework prioritizes mapping the extent and nature of evidence rather than evaluating the quality of included studies. The absence of a formal quality assessment, combined with considerable variation in study quality and methodological approaches, limits our ability to draw definitive conclusions about the strength of evidence or effectiveness of specific interventions.

The geographic distribution of included studies, with the majority conducted in the United States and limited representation from other migrant-receiving countries, may limit the generalizability of findings to diverse global contexts. Similarly, while the review captured diverse migrant populations, certain groups such as undocumented migrants, LGBTQ+ migrants, and migrants with disabilities were underrepresented in the reviewed literature, limiting our understanding of how to effectively serve these populations’ specific MHE needs. The variability in program success measures and outcome assessments across studies made it challenging to draw definitive comparisons or standardize best practices across different contexts and populations. Additionally, the review was limited to English-language publications, potentially excluding relevant programs and research published in other languages.

Building on these gaps, several critical research priorities emerge. Further research is needed to explore the long-term impact of MHE programs and their potential for scalability and sustainability. We concur with recommendations in the reviewed literature to assess effectiveness and increase generalizability by conducting larger randomized controlled trials (RCT). However, such trials must accommodate the complexity of culturally adapted interventions, employing designs sensitive to community dynamics and measurement equivalence across cultural groups.

Rigorous evaluation remains essential yet presents methodological challenges when outcomes are contingent on cultural fit. Measuring shifts in trust towards MHE program leaders or perceived relevance of program content demands tools sensitive to socio-cultural nuance rather than generic satisfaction surveys. Standardized metrics such as reduction in depression scores may miss subtler yet meaningful gains such as increased willingness to attend group sessions, more diverse sources consulted for support after intervention, or changes in collective coping repertoires and intergenerational dialogues about mental illness within families.

Research should also expand to focus on integration of arts-based practice in MHE programming. This aligns with the holistic approach emphasized in the reviewed literature, which aims to address the various and intersecting factors and realities that shape migrant mental health. A substantial amount of literature supports the use of arts-based therapy and community arts-based programming for mental health healing and promotion [77,78]. Demonstrated mental health benefits include increased empowerment, sense of social inclusion, self-esteem, rebuilding of identity, and self-discovery [77,78]. However, the literature exploring arts-inclusive MHE programming for immigrants and refugees is less documented. Research that does exist adds to the findings of psychological and social recovery benefits, such as reduced behavioral and emotional difficulties among undocumented immigrants [78,79].

### 4.2. Recommendations for Practice and Policy

Several insights and recommendations emerge from this review regarding best practices for the development and implementation of MHE programs for immigrants and refugees. First, programs should adopt holistic frameworks that address multiple determinants of health and wellbeing, highlighting both individual coping skills and community-level advocacy on systemic barriers such as racism, poverty, and immigration status. The literature emphasizes the need to invest in mental health interventions for marginalized populations that address social determinants, as this can improve not only mental but also physical and social health outcomes [58]. Strategies include tailoring coping skills content to the resettlement context and applying ecological models in programs to emphasize family and community interventions alongside individual skill-building, such as including conjoint family sessions to process migration stressors or engaging local faith and cultural groups in combating stigma and mobilizing social support.

Partnering with newcomer communities as program co-designers is essential to culturally adapt content and promote collective empowerment. This includes inviting community facilitators, using interpreters as needed, and translating materials. These strategies actively address social determinants through mechanisms that strengthen social support networks, empower communities toward social change, and create more accessible and effective paths to mental health care. Programs can build in sustainability and scalability by using train-the-trainer models, partnering with ethnic media for mental health literacy dissemination, and advancing newcomer leadership in research, service planning, and policy spheres.

Cultural safety also presupposes reflexivity among program administrators and facilitators including acknowledgment of potential biases shaped by lived experiences and training environments that privilege certain paradigms over others. Mental health providers in particular need competencies extending beyond clinical skills into civic and cultural domains so they can situate their roles within wider struggles for equity and inclusion. Training modules on these competencies could incorporate case-based learning developed in partnership with migrant communities themselves, ensuring curricula reflect genuine needs rather than outsider assumptions.

Institutional support through policy endorsement, secure funding, and workforce development is essential to maintain MHE program quality and scalability. Integration into mainstream mental health systems calls for workforce training in intercultural competencies, inclusion of culture brokers, and institutionalization of participatory governance structures that uphold empowerment and social justice principles.

Despite the limitations noted above, this review highlights a broad array of culturally sensitive MHE programs around the world from various literature sources, demonstrating both the need for and capability of reducing mental health inequities. The findings provide a foundation for future research and practice while illuminating critical areas where additional investigation and refinement are needed to optimize culturally adapted mental health education for migrant populations.

## 5. Conclusions

This scoping review of 28 studies and 4 grey literature sources demonstrates that culturally adapted MHE programs are acceptable, feasible, and potentially effective interventions for migrant populations. The evidence reveals that programs moving beyond surface-level adaptations to embrace deep cultural engagement, traditional knowledge integration, and community co-design show the most promise for meaningful impact. As global migration continues to reshape demographic landscapes, the imperative to develop mental health systems that are truly inclusive and equitable becomes increasingly urgent. The foundation for such systems exists in the innovative, culturally grounded programs identified in this review. Realizing their full potential requires sustained investment in rigorous research, policy reform that supports cultural safety frameworks, workforce development in intercultural competencies, and—most critically—the centering of migrant voices and leadership in all aspects of program design, implementation, and evaluation.

## Figures and Tables

**Table 1:** Key Characteristics of Included Studies.

Key Characteristics	Statistics	Details
Number of Studies	28	-
Age Range of Participants *	15–88	-
Country/Study Location	11	United States, United Kingdom, Turkey, Germany, Canada, Sweden, Netherlands, Kenya, Australia, Pakistan, Uganda
Program Content	-	2–14 training sessions, facilitated curriculum modules, group discussions, self-paced modules, and fotonovela
Cultural Adaptations	-	Linguistic adaptations, cultural symbolism and images, language adaptations, cultural values, cultural and religious stories, religious and spiritual activities, traditional/indigenous healing rituals and practices, arts-based activities, metaphors and proverbs, music, food, oral narratives and storytelling

* One study that included adolescents aged 15–17 alongside adults (age range 15–88 years) was retained given the relevance of the program model and the predominantly adult sample composition.

**Table 2:** Key Themes and Components of Culturally Adapted MHE Programs for Migrant Populations.

Themes	Key Components
Cultural Adaptation and Sensitivity	Surface-level adaptations (language, imagery); deep-level adaptations (values, worldviews, traditional practices); community involvement; culturally informed content
Addressing Unique Migration-Related Stressors and Challenges	Psychoeducation on PTSD, depression, anxiety; pre- and post-migration trauma; acculturation stress; discrimination and isolation; context-specific coping skills; system navigation support
Integration of Traditional and Western Approaches	Religious and spiritual practices; traditional healing ceremonies; cultural storytelling; indigenous explanatory models; traditional healers as co-facilitators; combined evidence-based and traditional techniques
Theoretical Frameworks and Evidence-Based Practices	CBT principles; CBPR approaches; cultural adaptation frameworks; trauma-informed care; social cognitive theory; intersectionality frameworks; evidence-based psychoeducation
Evaluation Methodologies	Pre-post designs; follow-up assessments; RCTs with control groups; mixed methods; culturally sensitive measures; assessment of literacy, stigma, symptoms, help-seeking behaviors
Application of Holistic Frameworks	Multiple wellbeing dimensions (physical, psychological, social, spiritual); intersectionality lens; social determinants of health; ecological approaches; family and community interventions
Community-Based Peer Support Models	Community partnership and co-design; peer and lay provider facilitation; community members as co-designers; cultural brokers; flexible and accessible delivery; train-the-trainer models; ethnic media partnerships

## Data Availability

No new data were created or analyzed in this study.

## Supplementary Materials

The following supporting information can be downloaded at: https://www.mdpi.com/article/10.3390/ijerph23010072/s1. Supplementary File S1: PRISMA-ScR Checklist.

## Abbreviations

The following abbreviations are used in this manuscript:

MHE	Mental health education
RCT	Randomized controlled trials
CBPR	Community-based participatory research
PTSD	Post-Traumatic Stress Disorder
PCC	Population–Concept–Context
CBT	Cognitive–Behavioral Therapy
PRISMA-ScR	Preferred Reporting Items for Systematic Reviews and Meta-Analyses extension for Scoping Reviews
LGBTQ+	Lesbian, Gay, Bisexual, Transgender, Queer/Questioning, and others

## Appendix A. Search Strategy

### Example Search Strategy

Ovid MEDLINE(R) ALL <1946 to 28 February 2024>

Date of search: 29 February 2024

1. exp “Emigrants and Immigrants”/or (immigrant* or immigration* or emigrant* or emigration* or incomer* or “in comer*” or “new comer*” or newcomer* or resettler* or “foreign born” or refugee* or “asylum seek*” or migrant*).mp.  91906
2. Mental Health/ed [Education]  490
3. mental health/and (education/ or teaching/)  438
4. exp Mental Disorders/ed [Education]  22
5. Mental Disorders/and (education/ or teaching/)  975
6. exp anxiety disorders/ed or exp obsessive-compulsive disorder/ed or exp phobic disorders/ed or exp “disruptive, impulse control, and conduct disorders”/ed or exp dissociative disorders/ed or exp mood disorders/ed or exp “bipolar and related disorders”/ed or exp depressive disorder/ed or exp personality disorders/ed or exp “schizophrenia spectrum and other psychotic disorders”/ed or exp psychotic disorders/ed or exp schizophrenia/ed or exp substance-related disorders/ed or exp alcohol-related disorders/ed or exp narcotic-related disorders/ed or exp “trauma and stressor related disorders”/ed or exp stress disorders, traumatic/ed or exp psychological trauma/ed or exp sexual trauma/ed  17
7. (Mental Health/ or exp Mental Disorders/ or exp anxiety disorders/ or exp obsessive-compulsive disorder/ or exp phobic disorders/ or exp “disruptive, impulse control, and conduct disorders”/ or exp dissociative disorders/ or exp mood disorders/ or exp “bipolar and related disorders”/ or exp depressive disorder/ or exp personality disorders/ or exp “schizophrenia spectrum and other psychotic disorders”/ or exp psychotic disorders/ or exp schizophrenia/ or exp substance-related disorders/ or exp alcohol-related disorders/ or exp narcotic-related disorders/ or exp “trauma and stressor related disorders”/ or exp stress disorders, traumatic/ or exp psychological trauma/ or exp sexual trauma/) and (educat* or teach* or learn* or literacy or literat* or school* or instruction* or guidance or train*).ab.  254397
8. ((“mental* health*” or “mental wellbeing” or “mental wellness” or “mental* ill*” or “mental disorder*” or “psychological health” or “psychological wellbeing” or “psychological wellness” or “psychological* ill*” or “psychological* disorder*” or “psychiatric illness*” or “psychiatric disorder*” or “psychiatric health” or “psychotic disorder*” or “psychotic condition*” or anxiety or phobia* or phobic or “panic disorder*” or “obsessive compulsive disorder*” or OCD or “stress disorder” or PTSD or “behavio?r disorder*” or “emotion* disorder*” or “oppositional defiant disorder” or ODD or “conduct disorder” or CD or “attention deficit hyperactivity disorder” or ADHD or bipolar or mania or manic or depression or depressive or psychotic or “mood disorder*” or suicid* or dissociative or dissociation or “depersonali?ation disorder” or “eating disorder*” or anorexi* or bulimi* or “binge eat*” or paranoia or paranoid or “personality disorder*” or schizophreni* or psychosis or “dysmorphic disorder*” or “self harm*” or “self mutilat*”) adj4 (educat* or teach* or learn* or inform* or literacy or literat* or school* or instruction* or guidance or prepar* or train*)).mp.  58807
9. ((grief or griev* or trauma) adj4 (educat* or teach* or learn* or inform* or literacy or literat* or school* or instruction* or guidance or prepar* or train*)).mp.  9263
10. or/2-9  299520
11. Cultural Competency/  6635
12. exp cultural diversity/  13325
13. ((cultur* adj3 (safe* or competen* or sensitiv* or appropriate* or specific or background* or adapt* or perspective* or experienc* or group* or identity or influenc* or tailor* or suitabl* or relevan* or conscious* or pluralism or divers*)) or transcultur* or multicultur* or multi-cultur* or crosscultur* or cross-cultur* or pluralism).mp.  149989
14. exp Complementary Therapies/ or (((aroma or art or “Bach flower” or bioelectromagnetic or biofield or bioresonance or chelation or colo?r or complementary or craniosacral or cupping or dance or horticultural or laughter or “mind body” or music or play or relaxation or sensory or soft tissue or spiritual or traditional or “trigger point” or touch or visualization) adj2 therap*) or (medicine* adj2 (complementary or alternative or integrative or traditional or functional or arabic or unani or ayurvedic or kampo or kanpo or Siddha or Tibetan or Mongolian or Chinese)) or acupressure or acupuncture or “autogenic training” or autosuggestion or anthroposophy or auriculotherapy or aromatherapy or biofeedback or “breathing exercises” or electroacupuncture or “diffuse noxious inhibitory control” or “faith healing” or herbalism or “historical eclecticism” or “holistic health” or homeopathy or integrative oncology or iridology or hypnosis or magic or ((musculoskeletal or osteopathic or chiropractic* or kinesiology) adj3 manipulations) or meditation or “mental healing” or meridians or mesotherapy or mindfulness or moxibustion or “manual lymphatic drainage” or naturopathy or neurofeedback or organotherapy or psychodrama or psychophysiology or phytotherapy or prolotherapy or qigong or reflexotherapy or reiki or radiesthesia or “role playing” or rolfing or seitai or shamani* or shiatsu or speleotherapy or “soul retrieval” or (traditional adj2 complementary) or (complementary adj2 alternative) or TCM or “tai ji” or “tai chi” or “therapeutic touch” or visualisation or yoga or witchcraft or “way? of knowing”).mp.  417776
15. ((linguistic* or language* or religious* or faith* or religion*) adj3 (safe* or competen* or sensitiv* or appropriate* or specific or background* or adapt* or perspective* or experienc* or group* or identity or influenc* or tailor* or suitabl* or relevan* or conscious* or pluralism or divers*)).mp.  29697
16. or/11-15  586349
17. 1 and 10 and 16  636

## Appendix B

**Table A1:** Program Characteristics of Included MHE Studies.

Article	Population	MHE Program	Program Content/Framework	Cultural Adaptation
Akhtar et al. (2021) [17]	Syrian refugees in camps and urban settings in Turkey	Group Problem Management Plus (PM+)	Psycho-educational program focusing on stress management, problem management, behavioral activation, social support strengthening	Arabic/Turkish; culturally adapted content, metaphors and illustrations; family engagement sessions; Arab facilitators; aligned with cultural values (family, religion, collective support)
Alvarez et al. (2024) [80]	Latina migrant population in US with ACEs.	Cuidándome (Self-Care)	9-module psycho-educational curriculum adapted from the DECIDE program teaching problem-solving for chronic disease self-management	Translated materials to Spanish; Used culturally relevant vignettes and examples.
Bentley et al. (2021) [39]	Muslim refugees from Islamic countries	Islamic Trauma Healing	Integrates empirically supported PTSD treatment components with Islamic principles and practices (e.g., narratives of prophets who experienced trauma	Developed collaboratively with Somali community members and Islamic leaders; Incorporates Islamic principles, practices and prophet narratives from Quran
Chow et al. (2010) [18]	Chinese and Tamil clients suffering from severe mental illness and their family members.	Multi-Family Psycho-education Group (MFPG)	Designed to teach families coping and problem-solving skills, increase knowledge about mental illness, and develop a support network, based on evidence that family interventions are critical for treatment of schizophrenia	Content modified to address culturally relevant issues like stigma, concerns about long-term medication use, etc. Conducted in participants’ native languages (Chinese, Tamil).
Ekblad (2020) [19]	New-comer, war exposed, low-educated Arabic/Somalian-speaking middle-aged mothers	Culturally relevant tailor-made group health promotion intervention.	Silove’s (1999) Adaptation and Development After Persecution and Trauma (ADAPT)	Tailored to cultural backgrounds of participants Conducted in participants’ native languages with interpreters.
Garabiles et al. (2019) [81]	Overseas Filipino workers (OFWs)	World Health Organization’s Step-by-Step program (e-mental health program).	A minimally guided self-help program, wherein a nonspecialist e-Helper provides technical support and assistance in accomplishing program activities through phone calls or text messaging for up to 20 min a week	Filipino values such as bayanihan (working together to help someone) and utang-na-loob (debt of gratitude) incorporated in the program stories and content. Characters’ personalities represented desirable Filipino values of family-orientation, warmth, care for others, sociability, and positive thinking.
Uribe Guajardo et al. (2018) [82]	Community-workers assisting Iraqi refugees on their resettlement	Mental Health Literacy Course adapted from the Mental Health First Aid training	Common mental illnesses, risk factors, barriers to help-seeking, and early intervention; Psychoeducation on depression, anxiety, and PTSD; Mental Health First Aid Action Plan	Culturally adapted resources on Iraqi patriarchal society, gender-specific communication barriers, and vignettes depicting Iraqi refugees with PTSD and depression for assessment
Gurung et al. (2020) [83]	Bhutanese refugees in the United States	Mental Health First Aid (MHFA) training	MHFA curriculum on mental illnesses (depression, anxiety, trauma, psychosis, substance use); 5-Step action plan for crisis intervention—(1) Assess the risk of suicide or harm, (2) Listen nonjudgmentally, (3) Give reassurance and information, (4) Encourage appropriate professional help, and (5) Encourage self-help and other support strategies	Bilingual English/Nepali delivery; Bhutanese trainer-led orientation; adapted case vignettes reflecting refugee experiences and migration stressors
Hendriks et al. (2024) [84]	Adult Arabic speaking refugees residing at asylum centers or municipalities in the Netherlands	BAMBOO—A culturally sensitive, strengths-based intervention	Positive psychology topics: strengths, emotions, relationships, gratitude, self-esteem; activities include art, meditation, goal setting	Surface adaptations: multilingual materials (in 6 languages), culturally appropriate images, local proverbs, symbol-based workbooks. Deep adaptations: Islamic principles, collectivistic values, gender-separated groups, movement-based exercises, interpreters, culturally framed stigma reduction
Hernandez & Organista (2013) [85]	Immigrant Latinas at risk for mental health concerns.	A Spanish-language fotonovela titled “Secret Feelings”	Fotonovela (entertainment–education) depicting Latina mother with depression; addresses symptoms, treatment options, stigma, and models help-seeking behaviors.	Fotonovela format; Spanish delivery by promotoras; culturally familiar group reading activities
Im et al. (2018) [36]	Somali refugees in Kenya and Somali community youth leaders in their mid-teens and early thirties.	Trauma-Informed Psychoeducation (TIPE)	Trauma psychoeducation including impacts of trauma (body, mind, relationships, spirituality); psychosocial competencies (coping, problem-solving, conflict management); Somali mental health terminology	Developed through collaboration with local Somali community organization; Peer-led by trained Somali youth leaders, assisted by community health counselors; applies cultural idioms of distress (welwel, murug, qaracan)
Im & Swan (2022) [35]	Refugee population	Interactive Training for Cross-Cultural Trauma-Informed Care (CC-TIC)	Trauma-sensitive curriculum covering refugee trauma, mental health issues, coping skills, cultural beliefs, acculturation stress; adapted from SAMHSA trauma guidelines	Cultural idioms of distress; mental health beliefs; community leader perspectives; acculturation stress.
Kiropoulos et al. (2011) [27]	CALD adult population in Australia. Study participants: Gr.	Multicultural Information on Depression Online (MIDonline)	Psychoeducation on depression: symptoms, case studies, diagnosis of depression, treatment options, finding bilingual professionals, stigma related to mental illness, and multilingual resources.	Program content in Greek, Italian, and English; Culturally relevant case studies representative of the target population
Koch et al. (2020) [23]	Young male Afghan refugees	Skills-Training of Affect Regulation–A Culture-sensitive Approach (STARC)	Transdiagnostic intervention focusing on emotion regulation: identifying emotions, regulation strategies (behavioral, cognitive, physiological), managing anger/sadness/anxiety	Incorporates cultural modifications to enhance program acceptability and effectiveness
Martinez et al. (2024) [21]	Filipino migrant domestic workers in the United Kingdom (UK).	Online mental health literacy (MHL) program called ‘Tara, Usap Tayo!’ (C’mon, Let’s Talk)	Psychoeducation curriculum based on content from WHO’s mhGAP, PM+, and the UK’s Adult IAPT, tailored and translated into Filipino.	Linguistically and culturally adapted for Filipino migrant domestic workers; integrates cultural values, beliefs, narratives, and language.
Morales et al. (2022) [24]	DACA (Deferred Action for Childhood Arrivals) recipients, undocumented immigrants, community leaders/organizers, mental health providers serving undocumented populations	Web-based tele-mentoring: “Strengthening Everyday Life Skills of DACA Recipients and Mixed-Status Families to Heal from Painful Emotions and Distress”	MHE focused on—Emotion regulation (mastery, coping, self-care) and distress tolerance (ACCEPTS, self-soothing, radical acceptance).	Program delivered via the Extension for Community Healthcare Outcomes (ECHO) DBT adapted with Latinx cultural values (familismo, personalismo, respeto, fatalismo); integrated dichos, spiritual practices, cultural foods, songs
Nogueira & Schmidt (2022) [22]	Latino immigrants	Project Esperanza: Mental health workshops	Mental health literacy topics: stigma, wellness, technology/cyberbullying, anxiety, depression, self-injury, suicide prevention, children’s and men’s mental health; focus on prevention and stigma reduction	Popular Education framework (Freire): Community stakeholder collaboration (practitioners, advocates, church staff, parents); co-creation of knowledge based on lived experiences; validates community strengths, wisdom, resilience
Omidian (2012) [30]	Afghan refugee teachers, their families and students	A psychosocial wellness training project	Psychosocial wellness, resilience, stress/emotion coping contextualized in Afghan culture; Gendlin’s focusing technique; children’s emotional development; balance of blessings	The activities were rooted in Afghani cultural elements (traditions, metaphors, poetry, Islamic texts); included collaborative workshops identifying indigenous wellness markers and coping mechanisms, Islamic values and texts
Ornelas et al. (2022) [86]	Spanish speaking immigrant Latina women	Amigas Latinas Motivando el Alma (ALMA)	Mindfulness-based curriculum (breath awareness, body scans, movement); emotional awareness and self-compassion; emphasis on social support and interconnectedness	Spanish delivery; Latino music, food, potluck meals, traditions, dichos; community co-developed; illustrated booklet by Latina immigrant artist; emphasized social support and interconnectedness
Poudel-Tandukar et al. (2021) [37]	Resettled Bhutanese adults aged 18 or older living in Western Massachusetts	Social and Emotional Well-being (SEW) intervention	Stress and coping theory (Lazarus & Folkman) and self-efficacy theory (Bandura); 5 weekly family sessions (health education, problem-solving, breathing, yoga); modules on stress management, communication, social networking, problem-solving, healthy family environment	Peer led by Bhutanese community interventionists; family-centered approach with daily home practice; culturally tailored health education and activities
Sabri et al. (2021) [26]	Immigrant women survivors of cumulative trauma.	Being Safe, Healthy, and Positively Empowered (BSHAPE)	Biopsychosocial and trauma-informed empowerment models; remote individual intervention via online platform and phone; strengths-based assessment, personalized modules (sexual/reproductive health, relationships, immigration, HIV/STIs, career, finance), 4-week mindfulness phone sessions, safety planning, resource linkage	Culturally grounded and Designed for Black immigrant women
Slewa-Younan et al. (2020) [31]	Arabic-speaking religious and community leaders in Southwestern Sydney working with refugee communities	Mental health literacy (MHL) training workshop tailored for Arabic-speaking religious and community leaders.	Mental health literacy workshop: recognition of refugee mental health issues, Australian treatment approaches, stigma reduction, role of leaders in help-seeking promotion	Arabic delivery; integrated Western treatment approaches with culturally/religiously informed practices (spiritual guidance, prayer); designed to complement religious/community leader roles
Slewa-Younan et al. (2020b) [33]	Arabic-speaking resettled refugees in Sydney with high distress levels	MHL program	MHL; wellbeing concepts, common mental disorders in refugees, Australian mental health system, self-help strategies (mindfulness, relaxation)	Arabic delivery by bilingual health educators/clinicians; culturally tailored content for refugee populations
Tol et al. (2018) [34]	South Sudanese refugees in northern Uganda	Self-Help Plus (SH+)—a guided self-help intervention developed by WHO	Self-Help Plus (SH+/WHO): based on principles of Acceptance and Commitment Therapy (ACT); stress management, problem-solving, behavioral activation, social support strengthening	Translated to Juba Arabic; culturally adapted language, metaphors, illustrations based on cognitive interviewing
Tran et al. (2014) [40]	Latino immigrants in US	ALMA (Amigas Latinas Motivando el Alma/Latina Friends Motivating the Soul),	Prevention-focused MHE focusing on- Mindfulness, self-compassion, stress/coping, social support strengthening	Spanish delivery; Latino music, food, art, migration stories, traditions; promotora-led; culturally tailored curriculum emphasizing social support and cultural connection
Uygun et al. (2020) [87]	Arabic-speaking adult Syrian refugees with high psychological distress in Turkey	Group Problem Management Plus (PM+)	Group Problem Management Plus (PM+): brief trans-diagnostic CBT-based psycho-edcuation; stress management, problem-solving, behavioral activation, social support strengthening	Culturally adapted for Syrian refugees; Arabic-speaking peer refugee facilitators; gender-matched groups
Weise et al. (2021) [88]	Adult asylum seekers in Germany experiencing mental distress.	The Tea Garden (TG)	Transdiagnostic psychoeducation: 4 modules covering trust-building, mental disorder symptoms, resources/self-care, treatment options	Native language delivery; gender-homogenous and language-homogenous groups; culturally relevant images, symbols, metaphors from participants’ backgrounds (nature, agriculture); body and animal analogies; interpreter support
Xin et al. (2011) [89]	Multiethnic adult refugee population.	Happy, Happy Community	Mental health promotion based on PRECEDE–PROCEED planning model; Health Belief Model (HBM); Social Support and Social Network Theory: 6 workshops on mental disorders/beliefs, acculturation, stress management, interpersonal relationships, job development, community safety; 12 support group discussions; depression screening; counseling referrals	Interpreters/cultural brokers with immigrant/refugee backgrounds; community-based needs assessment; culturally appropriate incentives (settlement resources); collaboration with similar programs; public health-social work partnership

## Appendix C

**Table A2:** Characteristics of MHE Programs Identified in Grey Literature.

Grey Literature	Population	MHE Program	Program Content/Framework	Cultural Adaptation
World Health Organization (WHO) (WHO, 2021, 2024) [28,29]	Populations affected by adversity	SELF-HELP PLUS (SH+)	Stress management program based on the principles of acceptance and commitment therapy	The program may be adapted to be used in different cultural contexts.
Hebrew Immigrant Aid Society (HIAS) (HIAS, 2021) [38]	Refugee and newcomers in the US	HIAS Mental Health and Psychosocial-support (MHPSS) Curriculum	Content includes Psychological first aid, cultural adjustment, family and community resilience. 9-week support group sessions, virtually or in person, with discussion, activities, exercises, and content.	Emphasizes facilitators be of refugee and immigrant background. Emphasizes value of community as a conduit for healing and sharing of cultural knowledge.
World Health Organization (WHO) [25]	Populations affected by adversity	Group Problem Management Plus PM+ Curriculum (stress and problem management, strengthening social support, and resilience).	Psychological intervention for individuals affected by adversity with emotional (depression, anxiety, stress, hopelessness, etc.) and practical problems (employment, housing, etc.). Group exercises, rituals, activities, and case examples.	Adapted for numerous cultures, languages and contexts; Highlights importance of being sensitive to and modifying content for different cultural norms, values, and religions.
Hong Fook Mental Health Association (Ho et al., 2002) [20]	Immigrant women from Cambodia, Korean, Chinese (Hong Kong, Mainland China, Taiwan), and Vietnamese communities	Women’s Holistic Health Peer Leadership Training Manual	Training manual to promote individual and collective empowerment for East and Southeast Asian immigrant peer Curriculum focused on 3 core components: understanding holistic health and mental health, exploring mental health perceptions and building communication and outreach skills.	Uses cultural symbols, food and artifacts, Sharing of personal histories and immigration experiences, collective book of stories. Program aided women to have increased understanding and awareness of mental health and illness and available culturally relevant resources; reduced stigma.

## Appendix D

**Table A3:** Evidence-Based Interventions with Multiple Implementations Across Migrant Populations.

Intervention	Studies (n)	Populations	Common Cultural Adaptations	Key Outcomes Reported
Mental Health First Aid (MHFA)	2	Bhutanese refugees (United States) Iraqi refugees (Australia)	Bilingual delivery with trained interpreters Adapted for Iraqi patriarchal society) Culturally adapted vignettes/case examples reflecting refugee experiences and migration stressors Community-member trainers from same cultural background Integration of cultural idioms of distress Gender-specific communication adaptations Faith leader and community worker involvement	Increased mental health knowledge and literacy Improved confidence in helping others experiencing mental health crises Reduced stigma toward mental health services Enhanced help-seeking intentions High program acceptability and feasibility
Problem Management Plus (PM+)	3	Syrian refugees (Turkey—2 studies) General populations affected by adversity (WHO grey literature)	Arabic/Turkish language translation; culturally adapted content, metaphors, and illustrations Arab/Syrian facilitators matched to participant culture and gender Family engagement sessions Aligned with cultural values (family, religion, collective support) Gender-matched groups Adapted to diverse cultural contexts, languages, norms, and religions	Reduced psychological distressDecreased anxiety& depression symptomsImproved problem-solving and coping skillsBehavioral activation and daily functioning improvementsStrengthened social supportGood feasibility, acceptability, and cultural appropriateness across contexts
Self-Help Plus (SH+)	2	South Sudanese refugees (Uganda) General populations affected by adversity (WHO grey literature)	Translated to Juba Arabic and adapted for multiple languages Culturally adapted language, metaphors, and illustrations based on cognitive interviewing ACT-based principles adapted to cultural contexts Illustrated self-help materials with culturally relevant imagery Minimal guidance format suitable for low-resource settings	Stress management improvementProblem-solving skill developmentBehavioral activation Social support strengtheningSuitable for diverse cultural contexts Self-guided format Increases accessibility
ALMA (Amigas Latinas Motivando el Alma / Latina Friends Motivating the Soul)	2	Spanish-speaking immigrant Latina women (United States—2 studies)	Spanish language delivery by promotoras (community health workers) Latino music, food, and potluck meals integrated into sessions Cultural traditions, dichos (sayings), and migration stories shared Community co-developed curriculum Illustrated booklet created by Latina immigrant artist Emphasis on social support, interconnectedness, and cultural connection Mindfulness and self-compassion adapted to Latina cultural context	Enhanced mindfulness and self-compassionImproved stress management and copingStrengthened social support networksIncreased cultural connection and sense of belonging High engagement and participant satisfactionCommunity empowerment

## References

[1] Kalich A., Heinemann L., Ghahari S. (2016). A Scoping Review of Immigrant Experience of Health Care Access Barriers in Canada. J. Immigr. Minor. Health.

[2] Thomson M.S., Chaze F., George U., Guruge S. (2015). Improving Immigrant Populations’ Access to Mental Health Services in Canada: A Review of Barriers and Recommendations. J. Immigr. Minor. Health.

[3] World Health Organization Mental Health of Refugees and Migrants: Risk and Protective Factors and Access to Care. https://iris.who.int/server/api/core/bitstreams/34945744-055a-4f3c-b931-d81a2bcf8f34/content.

[4] Scoglio A.A., Salhi C. (2020). Violence Exposure and Mental Health among Resettled Refugees: A Systematic Review. Trauma Violence Abus..

[5] Sigvardsdotter E., Vaez M., Hedman A.M., Saboonchi F. (2016). Prevalence of Torture and Other War-Related Traumatic Events in Forced Migrants: A Systematic Review. J. Rehabil. Torture Vict. Prev. Torture.

[6] Bustamante L.H.U., Cerqueira R.O., Leclerc E., Brietzke E. (2018). Stress, Trauma, and Posttraumatic Stress Disorder in Migrants: A Comprehensive Review. Braz. J. Psychiatry.

[7] Franks W., Gawn N., Bowden G. (2007). Barriers to Access to Mental Health Services for Migrant Workers, Refugees and Asylum Seekers. J. Public Ment. Health.

[8] Na S., Ryder A.G., Kirmayer L.J. (2016). Toward a Culturally Responsive Model of Mental Health Literacy: Facilitating Help-Seeking among East Asian Immigrants to North America. Am. J. Community Psychol..

[9] Fox S., Kramer E., Agrawal P., Aniyizhai A. (2022). Refugee and Migrant Health Literacy Interventions in High-Income Countries: A Systematic Review. J. Immigr. Minor. Health.

[10] Dingwall K.M., Puszka S., Sweet M., Nagel T. (2015). “Like Drawing into Sand”: Acceptability, Feasibility, and Appropriateness of a New e-Mental Health Resource for Service Providers Working with Aboriginal and Torres Strait Islander People. Aust. Psychol..

[11] Kirmayer L.J., Groleau D., Guzder J., Blake C., Jarvis E. (2003). Cultural Consultation: A Model of Mental Health Service for Multicultural Societies. Can. J. Psychiatry.

[12] Clelland N., Gould T., Parker E. (2007). Searching for Evidence: What Works in Indigenous Mental Health Promotion?. Health Promot. J. Aust..

[13] Peters M.D., Marnie C., Tricco A.C., Pollock D., Munn Z., Alexander L., McInerney P., Godfrey C.M., Khalil H. (2020). Updated Methodological Guidance for the Conduct of Scoping Reviews. JBI Evid. Synth..

[14] Peters M.D., Marnie C., Colquhoun H., Garritty C.M., Hempel S., Horsley T., Langlois E.V., Lillie E., O’Brien K.K., Tunçalp Ö. (2021). Scoping Reviews: Reinforcing and Advancing the Methodology and Application. Syst. Rev..

[15] Tricco A.C., Lillie E., Zarin W., O’Brien K.K., Colquhoun H., Levac D., Moher D., Peters M.D.J., Horsley T., Weeks L. (2018). PRISMA Extension for Scoping Reviews (PRISMA-ScR): Checklist and Explanation. Ann. Intern. Med..

[16] Bernal G., Jiménez-Chafey M.I., Domenech Rodríguez M.M. (2009). Cultural Adaptation of Treatments: A Resource for Considering Culture in Evidence-Based Practice. Prof. Psychol. Res. Pract..

[17] Akhtar A., Engels M.H., Bawaneh A., Bird M., Bryant R., Cuijpers P., Hansen P., Al-Hayek H., Ilkkursun Z., Kurt G. (2021). Cultural Adaptation of a Low-Intensity Group Psychological Intervention for Syrian Refugees. Intervention.

[18] Chow W., Law S., Andermann L., Yang J., Leszcz M., Wong J., Sadavoy J. (2010). Multi-Family Psycho-Education Group for Assertive Community Treatment Clients and Families of Culturally Diverse Background: A Pilot Study. Community Ment. Health J..

[19] Ekblad S. (2020). To Increase Mental Health Literacy and Human Rights among New-Coming, Low-Educated Mothers with Experience of War: A Culturally, Tailor-Made Group Health Promotion Intervention with Participatory Methodology Addressing Indirectly the Children. Front. Psychiatry.

[20] Ho M., Tse Y.H., Wong J.P.-H., Wong Y.-L.R. Women’s Holistic Health Peer Leadership Training Manual (For Health Educators & Trainers/Social Workers/Community Workers). Hong Fook Mental Health Association. Funded by the Ontario Women’s Health Council, March 2002. https://www.researchgate.net/publication/318402811_Womens_Holistic_Health_Peer_Training_Manual_March_2002.

[21] Martinez A.B., Lau J.Y., Morillo H.M., Brown J.S. (2024). ‘C’mon, Let’s Talk: A Pilot Study of Mental Health Literacy Program for Filipino Migrant Domestic Workers in the United Kingdom. Soc. Psychiatry Psychiatr. Epidemiol..

[22] Nogueira A.L., Schmidt I. (2022). “One Cannot Make It Alone”: Experiences of a Community Faith-Based Initiative to Support Latino Mental Health. Soc. Work Ment. Health.

[23] Koch T., Ehring T., Liedl A. (2020). Effectiveness of a Transdiagnostic Group Intervention to Enhance Emotion Regulation in Young Afghan Refugees: A Pilot Randomized Controlled Study. Behav. Res. Ther..

[24] Morales F.R., Rojas Perez O.F., Silva M.A., Paris M., Garcini L.M., Domenech Rodríguez M.M., Mercado A. (2022). Teaching DBT Skills to DACA Recipients and Their Families: Findings from an ECHO Program. Pract. Innov..

[25] World Health Organization (2018). Problem Management Plus (PM+): Individual Psychological Help for Adults Impaired by Distress in Communities Exposed to Adversity (Generic Field-Trial Version 1.1).

[26] Sabri B., Vroegindewey A., Hagos M. (2021). Development, Feasibility, Acceptability and Preliminary Evaluation of the Internet and Mobile Phone-Based BSHAPE Intervention for Immigrant Survivors of Cumulative Trauma. Contemp. Clin. Trials.

[27] Kiropoulos L.A., Griffiths K.M., Blashki G. (2011). Effects of a Multilingual Information Website Intervention on the Levels of Depression Literacy and Depression-Related Stigma in Greek-Born and Italian-Born Immigrants Living in Australia: A Randomized Controlled Trial. J. Med. Internet Res..

[28] World Health Organization (2021). Self-Help Plus (SH+): A Group-Based Stress Management Course for Adults.

[29] World Health Organization (2024). The Self-Help Plus (SH+) Training Manual: For Training Facilitators to Deliver the SH+ Course.

[30] Omidian P.A. (2012). Developing Culturally Relevant Psychosocial Training for Afghan Teachers. Intervention.

[31] Slewa-Younan S., Guajardo M.G.U., Mohammad Y., Lim H., Martinez G., Saleh R., Sapucci M. (2020). An Evaluation of a Mental Health Literacy Course for Arabic Speaking Religious and Community Leaders in Australia: Effects on Posttraumatic Stress Disorder Related Knowledge, Attitudes and Help-Seeking. Int. J. Ment. Health Syst..

[32] Poudel-Tandukar K., Jacelon C.S., Poudel K.C., Bertone-Johnson E.R., Rai S., Ramdam P., Hollon S.D. (2022). Mental Health Promotion among Resettled Bhutanese Adults in Massachusetts: Results of a Peer-Led Family-Centred Social and Emotional Well-Being (SEW) Intervention Study. Health Soc. Care Community.

[33] Slewa-Younan S., McKenzie M., Thomson R., Smith M., Mohammad Y., Mond J. (2020). Improving the Mental Wellbeing of Arabic Speaking Refugees: An Evaluation of a Mental Health Promotion Program. BMC Psychiatry.

[34] Tol W.A., Augustinavicius J., Carswell K., Brown F.L., Adaku A., Leku M.R., García-Moreno C., Ventevogel P., White R.G., Van Ommeren M. (2018). Translation, Adaptation, and Pilot of a Guided Self-Help Intervention to Reduce Psychological Distress in South Sudanese Refugees in Uganda. Glob. Ment. Health.

[35] Im H., Swan L.E. (2022). “We Learn and Teach Each Other”: Interactive Training for Cross-Cultural Trauma-Informed Care in the Refugee Community. Community Ment. Health J..

[36] Im H., Jettner J.F., Warsame A.H., Isse M.M., Khoury D., Ross A.I. (2018). Trauma-Informed Psychoeducation for Somali Refugee Youth in Urban Kenya: Effects on PTSD and Psychosocial Outcomes. J. Child Adolesc. Trauma.

[37] Poudel-Tandukar K., Jacelon C.S., Rai S., Ramdam P., Bertone-Johnson E.R., Hollon S.D. (2021). Social and Emotional Wellbeing (SEW) Intervention for Mental Health Promotion among Resettled Bhutanese Adults in Massachusetts. Community Ment. Health J..

[38] HIAS (2021). HIAS Mental Health and Psychosocial Support (MHPSS) Curriculum: Support Group Facilitator Guide.

[39] Bentley J.A., Feeny N.C., Dolezal M.L., Klein A., Marks L.H., Graham B., Zoellner L.A. (2021). Islamic Trauma Healing: Integrating Faith and Empirically Supported Principles in a Community-Based Program. Cogn. Behav. Pract..

[40] Tran A.N., Ornelas I.J., Perez G., Green M.A., Lyn M.J., Corbie-Smith G. (2014). Evaluation of Amigas Latinas Motivando El Alma (ALMA): A Pilot Promotora Intervention Focused on Stress and Coping among Immigrant Latinas. J. Immigr. Minor. Health.

[41] Ramsden I.M. (2002). Cultural Safety and Nursing Education in Aotearoa and Te Waipounamu. Ph.D. Thesis.

[42] Papps E., Ramsden I. (1996). Cultural Safety in Nursing: The New Zealand Experience. Int. J. Qual. Health Care.

[43] Curtis E., Jones R., Tipene-Leach D., Walker C., Loring B., Paine S.J., Reid P. (2019). Why Cultural Safety Rather than Cultural Competency Is Required to Achieve Health Equity: A Literature Review and Recommended Definition. Int. J. Equity Health.

[44] Kirmayer L.J. (2012). Rethinking Cultural Competence. Transcult. Psychiatry.

[45] Kirmayer L.J., Jarvis G.E. (2019). Culturally Responsive Services as a Path to Equity in Mental Healthcare. Healthcare Papers.

[46] Jorm A.F. (2012). Mental Health Literacy: Empowering the Community to Take Action for Better Mental Health. Am. Psychol..

[47] Kutcher S., Wei Y., Coniglio C. (2016). Mental Health Literacy: Past, Present, and Future. Can. J. Psychiatry.

[48] Resnicow K., Baranowski T., Ahluwalia J.S., Braithwaite R.L. (1999). Cultural Sensitivity in Public Health: Defined and Demystified. Ethn. Dis..

[49] Barrera M., Castro F.G. (2006). A Heuristic Framework for the Cultural Adaptation of Interventions. Clin. Psychol. Sci. Pract..

[50] Gone J.P., Trimble J.E. (2012). American Indian and Alaska Native Mental Health: Diverse Perspectives on Enduring Disparities. Annu. Rev. Clin. Psychol..

[51] Kirmayer L.J., Whitley R., Fauras V. (2009). Community Team Approaches to Mental Health Services and Wellness Promotion.

[52] Benish S.G., Quintana S., Wampold B.E. (2011). Culturally Adapted Psychotherapy and the Legitimacy of Myth: A Direct-Comparison Meta-Analysis. J. Couns. Psychol..

[53] Repper J., Carter T. (2011). A Review of the Literature on Peer Support in Mental Health Services. J. Ment. Health.

[54] Durie M. (1985). A Maori Perspective of Health. Soc. Sci. Med..

[55] Gone J.P. (2013). Redressing First Nations Historical Trauma: Theorizing Mechanisms for Indigenous Culture as Mental Health Treatment. Transcult. Psychiatry.

[56] Bhugra D., Becker M.A. (2005). Migration, Cultural Bereavement and Cultural Identity. World Psychiatry.

[57] Porter M., Haslam N. (2005). Predisplacement and Postdisplacement Factors Associated with Mental Health of Refugees and Internally Displaced Persons: A Meta-Analysis. JAMA.

[58] Kirkbride J.B., Anglin D.M., Colman I., Dykxhoorn J., Jones P.B., Patalay P., Pitman A., Soneson E., Steare T., Wright T. (2024). The Social Determinants of Mental Health and Disorder: Evidence, Prevention and Recommendations. World Psychiatry.

[59] World Health Organization Mental Health and Psychosocial Considerations During the COVID-19 Outbreak. https://www.who.int/publications/i/item/WHO-2019-nCoV-MentalHealth-2020.1.

[60] Murray L.K., Dorsey S., Haroz E., Lee C., Alsiary M.M., Haydary A., Weiss W.M., Bolton P. (2014). A Common Elements Treatment Approach for Adult Mental Health Problems in Low- and Middle-Income Countries. Cogn. Behav. Pract..

[61] Sijbrandij M., Kunovski I., Cuijpers P. (2016). Effectiveness of Internet-Delivered Cognitive Behavioral Therapy for Posttraumatic Stress Disorder: A Systematic Review and Meta-Analysis. Depress. Anxiety.

[62] Bronfenbrenner U. (1979). The Ecology of Human Development: Experiments by Nature and Design.

[63] Ungar M. (2011). The Social Ecology of Resilience: Addressing Contextual and Cultural Ambiguity of a Nascent Construct. Am. J. Orthopsychiatry.

[64] Wendt D.C., Gone J.P. (2018). Integrating Professional and Indigenous Therapies: An Urban American Indian Narrative Clinical Case Study. Couns. Psychol..

[65] Suite D.H., La Bril R., Primm A., Harrison-Ross P. (2007). Beyond Misdiagnosis, Misunderstanding and Mistrust: Relevance of the Historical Perspective in the Medical and Mental Health Treatment of People of Color. J. Natl. Med. Assoc..

[66] Israel B.A., Schulz A.J., Parker E.A., Becker A.B. (1998). Review of Community-Based Research: Assessing Partnership Approaches to Improve Public Health. Annu. Rev. Public Health.

[67] Wallerstein N.B., Duran B. (2006). Using Community-Based Participatory Research to Address Health Disparities. Health Promot. Pract..

[68] Castro F.G., Barrera M., Holleran Steiker L.K. (2010). Issues and Challenges in the Design of Culturally Adapted Evidence-Based Interventions. Annu. Rev. Clin. Psychol..

[69] Acarturk C., Kurt G., İlkkurşun Z., de Graaff A.M., Bryant R., Cuijpers P., Fuhr D., McDaid D., Park A.L., Sijbrandij M. (2024). Effectiveness of Group Problem Management Plus in Distressed Syrian Refugees in Türkiye: A Randomized Controlled Trial. Epidemiol. Psychiatr. Sci..

[70] Burchert S., Alkneme M.S., Alsaod A., Cuijpers P., Heim E., Hessling J., Hosny N., Sijbrandij M., Van’t Hof E., Ventevogel P. (2024). Effects of a Self-Guided Digital Mental Health Self-Help Intervention for Syrian Refugees in Egypt: A Pragmatic Randomized Controlled Trial. PLoS Med..

[71] de Graaff A.M., Cuijpers P., Twisk J.W.R., Kieft B., Hunaidy S., Elsawy M., Gorgis N., Bouman T.K., Lommen M.J.J., Acarturk C. (2023). Peer-Provided Psychological Intervention for Syrian Refugees: Results of a Randomised Controlled Trial on the Effectiveness of Problem Management Plus. BMJ Ment. Health.

[72] Spaaij J., Fuhr D.C., Akhtar A., Casanova L., Klein T., Schick M., Weilenmann S., Roberts B., Morina N. (2023). Scaling-Up Problem Management Plus for Refugees in Switzerland: A Qualitative Study. BMC Health Serv. Res..

[73] Purgato M., Carswell K., Tedeschi F., Acarturk C., Anttila M., Au T., Bajbouj M., Baumgartner J., Biondi M., Churchill R. (2021). Effectiveness of Self-Help Plus in Preventing Mental Disorders in Refugees and Asylum Seekers in Western Europe: A Multinational Randomised Controlled Trial. Psychother. Psychosom..

[74] Acarturk C., Uygun E., Ilkkursun Z., Carswell K., Tedeschi F., Batu M., Eskici S., Kurt G., Anttila M., Au T. (2022). Effectiveness of a WHO Self-Help Psychological Intervention for Preventing Mental Disorders Among Syrian Refugees in Turkey: A Randomised Controlled Trial. World Psychiatry.

[75] Tay A.K., Miah M.A.A., Khan S., Mohsin M., Alam A.N.M.M., Ozen S., Mahmuda M., Ahmed H.U., Silove D., Ventevogel P. (2021). A Naturalistic Evaluation of Group Integrative ADAPT Therapy (IAT-G) with Rohingya Refugees During the Emergency Phase of a Mass Humanitarian Crisis in Cox’s Bazar, Bangladesh. eClinicalMedicine.

[76] Arksey H., O’Malley L. (2005). Scoping Studies: Towards a Methodological Framework. Int. J. Soc. Res. Methodol..

[77] Van Lith T., Schofield M.J., Fenner P. (2013). Identifying the Evidence Base for Art-Based Practices and Their Potential Benefit for Mental Health Recovery: A Critical Review. Disabil. Rehabil..

[78] Stickley T., Duncan K. (2007). Art in Mind: Implementation of a Community Arts Initiative to Promote Mental Health. J. Public Ment. Health.

[79] Mateos-Fernández R., Saavedra J. (2022). Designing and Assessing of an Art-Based Intervention for Undocumented Migrants. Arts Health.

[80] Alvarez C., Sanchez-Roman M.J., Vrany E.A., Mata López L.R., Smith O., Escobar-Acosta L., Hill-Briggs F. (2024). Cuidándome: A Trauma-Informed and Cultural Adaptation of a Chronic Disease Self-Management Program for Latina Immigrant Survivors with a History of Adverse Childhood Experiences and Depression or Anxiety Symptoms. Cult. Divers. Ethn. Minor. Psychol..

[81] Garabiles M.R., Shehadeh M.H., Hall B.J. (2019). Cultural Adaptation of a Scalable World Health Organization e-Mental Health Program for Overseas Filipino Workers. JMIR Form. Res..

[82] Uribe Guajardo M.G., Slewa-Younan S., Kitchener B.A., Mannan H., Mohammad Y., Jorm A.F. (2018). Improving the Capacity of Community-Based Workers in Australia to Provide Initial Assistance to Iraqi Refugees with Mental Health Problems: An Uncontrolled Evaluation of a Mental Health Literacy Course. Int. J. Ment. Health Syst..

[83] Gurung A., Subedi P., Zhang M., Li C., Kelly T., Kim C., Yun K. (2020). Culturally-Appropriate Orientation Increases the Effectiveness of Mental Health First Aid Training for Bhutanese Refugees: Results from a Multi-State Program Evaluation. J. Immigr. Minor. Health.

[84] Hendriks T., Hassankhan A., de Jong J.T., van Woerkom M. (2024). Improving Resilience and Mental Well-Being among Refugees Residing at Asylum Centers in the Netherlands: A Pre-Post Feasibility Study. Ment. Health Prev..

[85] Hernandez M.Y., Organista K.C. (2013). Entertainment–Education? A Fotonovela? A New Strategy to Improve Depression Literacy and Help-Seeking Behaviors in at-Risk Immigrant Latinas. Am. J. Community Psychol..

[86] Ornelas I.J., Perez G., Maurer S., Gonzalez S., Childs V., Price C., Scott M.J., Amesty S., Rao D. (2022). Amigas Latinas Motivando El Alma: In-Person and Online Delivery of an Intervention to Promote Mental Health among Latina Immigrant Women. J. Integr. Complement. Med..

[87] Uygun E., Ilkkursun Z., Sijbrandij M., Aker A.T., Bryant R., Cuijpers P., Kiselev N., Morina N., Nissen A., Ventevogel P. (2020). Protocol for a Randomized Controlled Trial: Peer-to-Peer Group Problem Management Plus (PM+) for Adult Syrian Refugees in Turkey. Trials.

[88] Weise C., Grupp F., Reese J.P., Schade-Brittinger C., Ehring T., Morina N., Knaevelsrud C., Kamp-Becker I., Stoll M., Jelinek L. (2021). Efficacy of a Low-Threshold, Culturally Sensitive Group Psychoeducation Programme for Asylum Seekers (LoPe): Study Protocol for a Multicentre Randomised Controlled Trial. BMJ Open.

[89] Xin H., Bailey R., Jiang W., Aronson R., Strack R. (2011). A Pilot Intervention for Promoting Multiethnic Adult Refugee Groups’ Mental Health: A Descriptive Article. J. Immigr. Refug. Stud..

